# Corticosteroid‐responsive narcolepsy type II after COVID‐19: A relevant differential diagnosis of post‐COVID syndrome (a case report)

**DOI:** 10.1111/jsr.14406

**Published:** 2024-12-02

**Authors:** Erika C. S. Künstler, Solveig Menrad, Isabelle Utech, Kathrin Finke, Sven Rupprecht

**Affiliations:** ^1^ Department of Neurology Jena University Hospital Jena Germany; ^2^ Interdisciplinary Centre for Sleep and Ventilatory Medicine Jena University Hospital Jena Germany

**Keywords:** case report, excessive daytime sleepiness, post‐COVID, remission, symptomatic narcolepsy

## Abstract

Excessive daytime sleepiness is a possible symptom of post‐COVID syndrome and is also the cardinal symptom of narcolepsy, a rare life‐long sleep disorder with a possible autoimmune background. Recent reports indicate that COVID‐19 infection may trigger narcolepsy. However, it remains unclear how best to identify and treat such cases. A 25‐year‐old male developed daytime sleepiness after COVID‐19 infection. A diagnosis of narcolepsy type II was made based on pathologically shortened sleep latencies in polysomnography and multiple sleep latency tests (MSLT) together with several sleep‐onset REM‐sleep periods (SOREMs). Pupillography and neuropsychological testing revealed reduced alertness levels. Hypocretin levels in the cerebrospinal fluid were borderline. Based on the postulated autoimmune background of narcolepsy, we performed an intravenous high‐dose corticosteroid pulse therapy with methylprednisolone. Narcoleptic symptoms immediately and consistently remitted after the corticosteroid pulse. Follow‐up after 4 months revealed normalisation of sleep latencies, no further SOREMs in the MSLT, and increased alertness in pupillography and neurocognitive testing. No further wakefulness promoting drug therapy was required. Narcolepsy should be considered in the differential diagnosis of post‐COVID syndrome with leading symptoms of daytime sleepiness. Furthermore, immunosuppressive therapy may offer a treatment option in managing an otherwise lifelong disorder in select cases.

## 1 INTRODUCTION

A common symptom in post‐COVID syndrome is excessive diurnal sleepiness (EDS, Merikanto et al., 2023). However, EDS may also point to a central disorder of hypersomnolence, including narcolepsy (American Academy of Sleep Medicine, 2014). Narcolepsy – linked to an autoimmune‐mediated loss of hypocretinergic cells (Bassetti et al., 2019) – is typically marked by EDS, sleep paralysis, cataplexy, and hypnagogic/hypnopompic hallucinations, and is accompanied by shortened sleep latencies and sleep‐onset REM periods (SOREMs, American Academy of Sleep Medicine, 2014). The increased incidence of narcolepsy following previous viral pandemics initially fuelled concern of a similar outcome following the SARS‐CoV‐2 pandemic (Mignot & Black, 2020). Fortunately, only isolated cases have been reported (Deshpande et al., 2022; Roya et al., 2023). However, these prior publications have either not reported treatment (Deshpande et al., 2022), or suggest that treatment may be difficult (Roya et al., 2023).

We present a case of a young man who developed narcolepsy type II after a SARS‐CoV‐2 infection (PCR confirmed). His symptoms drastically improved following an individual curative immunosuppressive attempt using a single corticosteroid pulse with methylprednisolone. To the best of our knowledge, this is the first case in which an individual curative attempt was made using immunosuppressive therapy following narcolepsy after COVID‐19 infection. As narcolepsy would otherwise present a chronic disorder necessitating life‐long medication, this case report offers an attractive alternative in select cases, and highlights the need to regard narcolepsy as a differential diagnosis of post‐COVID syndrome with EDS as the leading symptom.

## 2 CASE PRESENTATION

### 2.1 Initial case presentation

A 25‐year‐old male patient was referred to our sleep laboratory via our post‐COVID outpatient clinic for diagnostic assessment of daytime sleepiness (Epworth Sleepiness Scale (ESS) 11/24 points). The patient's medical history prior to the COVID‐19 infection in December 2020 was completely unremarkable. The infection (confirmed following NICE guidelines) progressed into post‐COVID syndrome with hyposmia, post‐exertional malaise, sleepiness, minimal cognitive impairment with reduced alertness (see Table 1), and nightly headaches. The patient developed myocarditis (a known complication of COVID‐19) following infection, and was treated with 2.5 mg ramipril during both the initial presentation and at follow‐up 12 months later. As a shift worker, he reported excessive sleepiness, especially when working early shifts. He negated classical narcoleptic symptoms such as an imperative urge to sleep in monotonous situations, cataplexy, hallucinations, and sleep paralysis. However, he had very vivid and intense dreams since the infection. An increase in dream recall frequency and nightmare frequency has been reported as accompanying a COVID‐19 infection (Scarpelli et al., 2022).

**TABLE 1:** Summary of findings at initial presentation and at follow‐up 12 months later, 4 months after having undergone immunosuppressive therapy.

	At initial presentation	At follow‐up, 12 months later
Multiple sleep latency test (MSLT)		
Averaged sleep latency	5 min (range: 1–11 min)	12 min (range: 6–20 min)
Number of trials in which the patient slept	5 of 5	3 of 5
Number of SOREMs	3	0
Polysomnographic data (averaged over two nights pre‐intervention and three nights post‐intervention)		
Time in bed (TIB)	8 h 52 min (range: 8 h 37 min–9 h 06 min)	8 h 33 min (range: 8 h 10 min–9 h 07 min)
Total sleep time (TST)	8 h 31 min (range: 8 h 17 min–8 h 45 min)	7 h 55 min (range: 7 h 49 min–7 h 58 min)
Sleep efficiency (%)	96.1% (range: 96.0%–96.1%)	92.7% (range: 85.7%–97.6%)
Sleep latency (minutes)	2 min (range: 2–2.5 min)	3 min (range: 1–5 min)
REM latency (hours/minutes)	35 min (range: 14–56 min)	37 min (range: 9–53 min)
Number of SOREMs	1	1
Stage 1 (% of TST)	8.1% (range: 6.8%–9.3%)	8.9% (range: 7.7%–11.1%)
Stage 2 (% of TST)	52.5% (range: 49.9%–55.1%)	40.6% (range: 38.3%–43%)
Stage 3 (% of TST)	17.4% (range: 17.3%–17.4%)	21.1% (range: 18.6%–25.9%)
REM sleep (% of TST)	22.1% (range: 20.7%–23.4%)	29.6% (range: 28%–30.8%)
Arousal/awakening index (/hour)	7.5/h (range: 6.7–8.3/h)	9.2/h (range: 4.8–12.2/h)
Number of sleep‐stage transitions	99 (range: 94–104)	99 (range: 86–118)
Apnea/Hypopnea index (AHI; ref.: 0–5/h)	0.1/h (range: 0.1–0.1/h)	0.8/h (range: 0.1–1.3/h)
Periodic leg movement index (PLM; ref.: 0–5/h)	4.2/h (range: 2.8–5.6/h)	4.3/h (range: 3.0–6.0/h)
Pupillary unrest index (mm/min; ref.: normal: ≤6.6; borderline: 6.7–9.8; pathological: ≥9.9)		
11.30 a.m.	11.8	8.4
13.30 p.m.	13.9	7.1
15.30 p.m.	13.6	7.7
17.30 p.m.	11.1	7.6

Neuropsychological testing (conducted using the German version of "test of attentional performance (Psytest)")			
		At post‐COVID outpatient clinic (2 months before initial presentation)	At follow‐up (12 months after initial presentation)
Tonic alertness ("Intrinsic alertness" subtest)	Median (ms)	283 (percentile: 8)	229 (percentile: 38)
	SD (ms)	27 (percentile: 62)	26 (percentile: 66)
Phasic alertness ("Phasic arousal" subtest)	Median (ms)	271 (percentile: 10)	238 (percentile: 24)
	SD (ms)	48 (percentile: 18)	32 (percentile: 46)
Vigilance ("Visual: moving bar" subtest)	Median (ms)	‐	636 (percentile: 34)
	SD (ms)		96 (percentile: 79)
	Missed targets		0 (percentile: >93)
	Errors		0 (percentile: >76)

Abbreviations: REM, rapid eye movement; SOREM, sleep‐onset REM sleep; TST, total sleep time.

### 2.2 Initial findings

**FIGURE 1:**
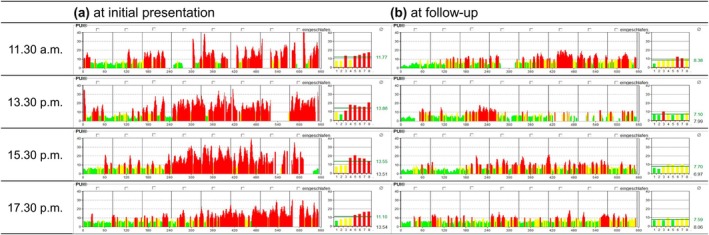
Pupillographies showing the pupillary unrest index before (a) and after (b) intervention.

An extensive diagnostic workup was conducted in the Interdisciplinary Centre for Sleep and Ventilatory Medicine of the Jena University Hospital. Physical examination, routine blood tests, and cranial magnetic resonance imaging were unremarkable. SOREMs were observed in three of four trials of the multiple sleep latency test (MSLT), and during polysomnography of the following night. A shortened average sleep latency of 5 min in the MSLT indicated pathological sleep pressure (Table 1). Consistently, repeated pupillographies showed reduced tonic alertness (Figure 1a). Both polysomnographies showed phasic missing muscle atonia in REM sleep. Overall, polysomnograpic recordings suggested the absence of comorbid sleep disorders. Hypocretin‐1 levels were assessed via lumbar puncture, revealing a borderline value of 170 pg/mL in the cerebrospinal fluid (^125^I radioimmunoassay kit (Phoenix Pharmaceuticals, Belmont, CA, USA); Ripley et al., 2001, reference: normal: ≥200 pg/mL; narcolepsy type I: ≤110 pg/mL) and no signs of inflammation. Due to the polysomnographic abnormalities and the preserved hypocretin values, a diagnosis of narcolepsy type II was made. Tentatively, this diagnosis may also be considered to be symptomatic narcolepsy type II following SARS‐CoV‐2 infection. Wake‐promoting therapy (modafinil, 2 × 100 mg/day) was initiated.

### 2.3 Therapeutic intervention

Due to delays in the external assessment of hypocretin‐1 levels, therapeutic intervention was performed 8 months after diagnosis. Most of the post‐COVID symptoms had remitted, but daytime sleepiness and vivid dreams persisted and were subjectively comparable to the initial presentation. We interpreted the borderline hypocretin levels as indicative of autoimmune‐induced damage of the hypocretinergic system. Thus, we hypothesised that immune‐suppressive therapy, as an individual curative trial, could halt the autoimmune process, possibly leading to a remission of narcoleptic symptoms. We performed a single high‐dose corticosteroid pulse with methylprednisolone (1 g/day given intravenously over 3 days, adapted from pulse steroid therapy in multiple sclerosis relapses). Simultaneously, 40 mg/day pantoprazol was given to reduce gastric side effects, whilst tinzaparin natrium 3500 IE was injected as thrombosis prophylaxis. The patient reported marked improvement in EDS within a few days after treatment. Modafinil was discontinued, as it was no longer required.

### 2.4 Follow‐up findings

Four months after the corticosteroid pulse, the patient presented for follow‐up. He reported a sustained improvement in his sleepiness (ESS score had decreased from 11 to 7 points), no further difficulties with shift work, and no intense dreaming. Objectively, the MSLT showed a remarkable improvement, with a normalisation of sleep latencies and no further SOREMs. Repeated pupillographies indicated a pronounced improvement in tonic alertness (Figure 1b). Neuropsychologically, the patient had normal performance in tasks of both alertness and vigilance under monotonous conditions.

Polysomnographies revealed a single SOREM, and a notable reduction in total sleep time. Muscle atonia in REM sleep improved, although some periods of increased phasic activity persisted. Additionally, the proportion of deep sleep increased, possibly explaining the subjective perception of sleep as being more restorative. See Table 1 for a side‐by‐side comparison of findings from both the initial presentation and follow‐up.

## 3 DISCUSSION

This case report objectively documents the remittance of narcoleptic symptoms arising after SARS‐CoV‐2 infection following a curative immunosuppressive attempt using a single intravenous high‐dose corticosteroid pulse. Overall, the patient's symptoms showed drastic improvement, and wakefulness‐promoting medication was no longer needed. This case highlights three key points: (1) COVID‐19 may trigger autoimmune destruction of hypocretinergic neurons; (2) immunosuppressive therapy may potentially offer an effective therapeutic alternative in select cases; and (3) both narcolepsy types I and II should be excluded as a differential diagnosis in patients with post‐COVID syndrome presenting with EDS.

The autoimmune pathogenesis of narcolepsy has been widely discussed (Bassetti et al., 2019; Giannoccaro et al., 2021). Initially, patient data had suggested that regulatory CD4^+^ T cells specifically target antigens expressed by hypocretinergic neurons. However, animal data and, later, patient data showed that it is rather the cytotoxic CD8^+^ T mediated immune response that causes hypocretinergic damage (Kornum & Jennum, 2020). Phenotypically, only a near‐to or total loss of hypocretinergic neurons results in >75% reduction of hypocretin‐1 levels in the CSF, and the development of the full clinical picture of narcolepsy, whilst a partial loss may cause daytime sleepiness without cataplexy and normal or reduced hypocretin‐1 levels in the CSF (Bassetti et al., 2019). As the loss of hypocretinergic cells has been suggested to be ongoing at symptom onset (Kornum & Jennum, 2020), we hypothesised that potential hypocretinergic cell loss in our patient may have led to the EDS and polysomnographic and MSLT anomalies. The full remittance of EDS and drastic reduction of SOREMs following corticosteroid therapy in our case further supports this autoimmune pathogenesis of narcolepsy. However, to what extent the immunological mechanisms after COVID‐19 infection resemble those seen in "classical" narcolepsy remains unclear.

Given the pathophysiology of narcolepsy, prompt immunosuppressive/immunomodulatory therapy seems to offer an attractive therapeutic option. Unfortunately, previous attempts highlight the complexity of putting this knowledge into practice. For example, intravenous immunoglobulin therapy (IVIg) attempts have mostly not been effective, also in cases of narcolepsy following H1N1 infection (Kornum & Jennum, 2020). Only when given directly following symptom onset has there been some amelioration (Dauvilliers et al., 2004). A caveat here is that extremely abrupt symptom onset may indicate irrevocable hypocretinergic damage (Lecendreux et al., 2003). The lack of efficacy of IVIg may be due to this treatment not directly affecting the CD8^+^ mediated cytotoxic immune response (Norris et al., 2020). Given that recent findings suggest that CD8+ T‐cells are involved in the disease development of narcolepsy type I, and given that corticosteroid therapy affects CD8+ T‐cells, this line of therapy may be more effective. Indeed, one case report showed that IVIg pulse therapy combined with a 3‐week prednisolone therapy (1 g/kg/day IVIg, then 1.3 mg/kg/day of prednisolone over 3 weeks) led to a dramatic improvement (Lecendreux et al., 2003). Finally, in addition to the timing and type of therapy being important, the therapy mode is also vital. Even though our patient did not fulfil the diagnostic criteria of narcolepsy type I, we performed a high dose pulse therapy as some evidence shows that lower doses may not be as efficacious, at least in the treatment of type I. For example, a 3‐week medium dose oral corticosteroid trial (1 mg/kg/day of prednisone) was ineffective, despite therapy having been initiated 2 months after symptom onset (Hecht et al., 2003). To what extent high dose intravenous corticosteroid pulse therapy is superior to oral continuous weight‐adjusted low/medium dosage therapy regimes, as given in other autoimmunological conditions, warrants further clarification.

While narcolepsy is considered a non‐phasic chronic disease, we cannot rule out a spontaneous remission. Whether spontaneous remission of narcolepsy is even possible remains unclear. Longitudinal studies have shown that the symptoms of EDS can subjectively fluctuate greatly over time (Büchele et al., 2018). However, it is unknown whether possible fluctuations in symptoms are sustained, and/or measurable via polysomnography. In the present case, a spontaneous remission seems highly unlikely given the persistence of daytime sleepiness and vivid dreaming up until the therapeutic intervention, and given the direct and sustained remittance of these symptoms after the corticosteroid pulse therapy. This case report has several further limitations. Firstly, no vigilance task was performed during the initial assessment, rendering the comparison of neuropsychological abilities pre‐ and post‐intervention incomplete. Secondly, EDS was not re‐evaluated prior to the start of the immunosuppressive therapy, which would have allowed a more precise quantification of sleepiness over time. Finally, a second lumbar puncture was not performed at follow up. This would have allowed invaluable inferences about hypocretinergic cell functioning to be made.

Finally, it must be noted that narcolepsy type II is an entity with unclear aetiology, which lacks specific and sensitive diagnostic markers, as SOREMs may occasionally be observed in healthy adults (Fronczek et al., 2020). However, the four SOREMs observed during the initial diagnosis of this case is very likely indicative of pathology beyond what can be expected of post‐COVID. Whether the narcolepsy reported here is a “classical” type II case, or whether it is rather a case of symptomatic type II narcolepsy remains unclear. Moreover, the lack of overt narcoleptic symptoms – apart from EDS – in our patient highlights a conundrum in the identification of similar patients, suggesting that narcolepsy following COVID‐19 may be under‐diagnosed. This implicates that more rigorous diagnostic workup of post‐COVID patients with a leading symptom of EDS is necessary to identify those patients who developed a post‐viral central disorder of hypersomnolence after COVID‐19 infection, and would potentially benefit from a curative immunosuppressive treatment.

## AUTHOR CONTRIBUTIONS

**Erika C. S. Künstler:** Writing – original draft; funding acquisition; methodology; conceptualization; formal analysis; data curation; project administration; writing – review and editing; validation; investigation; software. **Solveig Menrad:** Writing – original draft; methodology; conceptualization; formal analysis; data curation; project administration; writing – review and editing; visualization; validation; investigation; software. **Isabelle Utech:** Writing – review and editing; methodology; data curation; investigation; software; validation. **Kathrin Finke:** Supervision; project administration; conceptualization; writing – review and editing; methodology. **Sven Rupprecht:** Supervision; project administration; conceptualization; writing – review and editing; validation; methodology; resources; investigation; funding acquisition; formal analysis.

## CONFLICT OF INTEREST STATEMENT

All authors report no conflict of interest.

## Data Availability

The data that support this case report are available upon reasonable request from the corresponding author, but are not publicly available due to privacy restrictions.
